# Safety and Efficacy of Telehealth Medication Abortions in the US During the COVID-19 Pandemic

**DOI:** 10.1001/jamanetworkopen.2021.22320

**Published:** 2021-08-24

**Authors:** Ushma D. Upadhyay, Leah R. Koenig, Karen R. Meckstroth

**Affiliations:** 1 Department of Obstetrics, Gynecology, and Reproductive Sciences, University of California, San Francisco; 2 Department of Epidemiology and Biostatistics, University of California, San Francisco

## Abstract

This cohort study examines the safety and efficacy of fully remote, asynchronous medication abortion care during the COVID-19 pandemic.

## Introduction

Early in the COVID-19 pandemic, medication abortion, which typically includes mifepristone (ie, progesterone receptor antagonist) and misoprostol (ie, prostaglandin), gained prominence because it can be provided without physical contact. The American College of Obstetricians and Gynecologists and other professional organizations quickly endorsed telehealth and no-test abortion care.^1^ These protocols omit Rh testing^2^ and use patient history, rather than routine ultrasonography, to assess pregnancy duration and screen for ectopic pregnancy risks.^3,4^

To mitigate potential risk of complications, US Food and Drug Administration (FDA) Risk Evaluation and Mitigation Strategy (REMS) require that mifepristone be dispensed in a medical office, clinic, or hospital, prohibiting dispensing from pharmacies. Between a federal judge’s ruling that suspended enforcement of this requirement in July 2020 and a reversal by the Supreme Court in January 2021, clinicians could offer medication abortion via telehealth and dispense from mail-order pharmacies where not prohibited by state law. During this period, a virtual clinic called Choix began providing medication abortions in California. We assessed safety and efficacy outcomes of a telehealth medication abortion model, which could inform the FDA’s decision regarding removal of the REMS.

## Methods

This retrospective cohort study was approved by the institutional review board at the University of California, San Francisco, and informed consent was waived because the research presented no more than minimal risk. We followed the Strengthening the Reporting of Observational Studies in Epidemiology (STROBE) reporting guideline for cohort studies.

We examined the safety and efficacy of fully remote, asynchronous medication abortion care using a published protocol.^3^ Eligibility criteria, assessed using an online form, included pregnancy duration of less than 70 days (by history, including date of last menstrual period, or by ultrasonography) and no contraindications to mifepristone or misoprostol. Nurse practitioners reviewed the form within 24 hours and referred patients with unknown last menstrual period date or ectopic pregnancy risk factors for ultrasonography to confirm eligibility. A mail-order pharmacy delivered medications to eligible patients. The protocol involved 3 follow-up contacts: confirmation of medication administration, a 3-day assessment of expulsion and pregnancy symptoms, and a 4-week home pregnancy test. Follow-up interactions were conducted by text, secure messaging, or telephone. At each scheduled follow-up, clinicians made up to 4 attempts to contact patients.

In accordance with Choix’s privacy policy, the service shared deidentified health record data for all patients with our research team. Efficacy was assessed as complete abortion without additional intervention (aspiration or other procedure, surgery, >1600 µg of misoprostol, or continuing pregnancy) among those with a known abortion outcome from the 3-day or 4-week follow-up. Safety was assessed by any major adverse event, including blood transfusion, abdominal surgery, hospital admission, or death. We estimated outcomes as percentages and calculated 95% CIs for key rates in Stata version 15.1 (StataCorp).

## Results

The service provided abortion care for 141 patients between October 2020 and January 2021. Mean (SD) participant age was 29 (7) years, 81 (57%) had pregnancy durations 42 days or less, and 24 (17%) had screening ultrasonography (Table 1). At least 1 follow-up contact was made for 128 patients (91%), and abortion outcomes were collected for 110 patients (86%). Among the 110 patients with outcome data, 105 (95%) had a complete abortion without intervention. Five patients (5%) required medical care to complete the abortion, 2 of whom were treated in emergency departments. No patients reported any major adverse events (Table 2).

**Table 1.** Description of Patient Characteristics

Characteristic	Total, No. (%), (n = 141)
Age, mean (SD)	28.5 (6.7)
Had ultrasonography before abortion	24 (17.0)
Pregnancy duration, d	
≤42	81 (57.4)
43-56	43 (30.5)
57-70	17 (12.1)
Rh type	
Positive	67 (47.5)
Unknown	60 (42.6)
Negative	14 (9.9)
Received Rh immune globulin	0
Any follow-up information collected	128 (90.8)

**Table 2.** Abortion Safety and Efficacy Outcomes

Follow-up data	Total, No./total No. (%), [n = 128]
Efficacy	
Outcome unknown^a^	18/128 (14.1)
Outcome known	110/128 (85.9)
Complete, No./total No. (% [95% CI])	105/110 (95.5 [91.6-99.3])
Complete by test^b^	88/110 (80.8)
Complete by history^c^	17/110 (15.5)
Required additional medical intervention for completion, No./total No. (% [95% CI])^d^	5/110 (4.5 [0.7-8.4])
Safety, No./total No. (% [95% CI])	
Confirmed or suspected ectopic pregnancy	0/128 (0 [0.0-2.3])
Any major adverse event^e^	0/128 (0 [0.0-2.3])

^a^ Ten patients confirmed initial administration of medications and were subsequently lost to follow-up. Eight patients had inconclusive symptoms or test results and were subsequently lost to follow-up.

^b^ Confirmed complete by 4-week urine pregnancy test.

^c^ Presumed complete by symptom assessment 3 days after medications.

^d^ Overall, 3 patients had an aspiration or dilation and curettage (2 treated in emergency department), and 2 patients required 2400 µg of misoprostol.

^e^ No patients had a major adverse event defined as blood transfusion, surgery, hospital admission, or death.

## Discussion

These results represent some of the earliest data on new telehealth abortion clinics in the US. This 95% efficacy rate is similar to in-person provision^5^ and recent international studies of telehealth for medication abortion.^6^

In April 2021, the FDA paused enforcement of the REMS for the duration of the COVID-19 pandemic, allowing clinics and pharmacies to mail mifepristone once again. The FDA is now considering permanently removing the REMS. This study is small with some loss to follow-up, and thus some adverse events and ongoing pregnancies may have been undetected. However, it reflects real-world data, which increases generalizability. This study provides preliminary evidence that suggests medication abortion care, administered by telehealth and delivered via mail, is feasible, safe, and efficacious.
